# Direct visualization system combined with endoscopic retrograde cholangiopancreatography for treatment of choledocholithiasis complicated by multiple gallstones

**DOI:** 10.1055/a-2376-5834

**Published:** 2024-09-04

**Authors:** Wei Zhang, Lichao Zhang, Sen-Lin Hou

**Affiliations:** 1 71213 Biliopancreatic Endoscopic Surgery, The Second Hospital of Hebei Medical University, Shijiazhuang, China

Direct visualization system combined with endoscopic retrograde cholangiopancreatography for treatment of choledocholithiasis complicated by multiple gallstones.

Currently, the first-line treatment for multiple gallstones with choledocholithiasis is endoscopic retrograde cholangiopancreatography (ERCP) combined with cholecystectomy. However, many patients do not proceed with cholecystectomy after ERCP. In particular, young patients often have a strong preference for preserving the gallbladder. We developed a method to remove gallstones by utilizing the natural cavity pathways of the human body.

A 30-year-old woman was hospitalized with choledocholithiasis and gallstones ( Fig. 1 ) for gallbladder-preserving lithotomy after ERCP. First, we performed ERCP to remove the common bile duct (CBD) stones. Then, we inserted a plastic stent in the CBD to prevent obstructive jaundice after the placement of a fully covered metal stent in the cystic duct of the gallbladder. Next, a fully covered metal stent with a diameter of 10 mm and a length of 12 cm was placed in the cystic duct of the gallbladder ( Fig. 1 ). Three days later, we performed ERCP-based cholecystolithotomy assisted by the eyeMax direct visualization system. Finally, the stents were gradually removed, the gallbladder cavity was irrigated ( Fig. 1 ), and an external drainage tube was placed inside the gallbladder. After 2 days, the tube was removed. The patient experienced no complications. We followed up the patient for 10 months, during which no gallstones were found on abdominal ultrasound ( Video 1 ).

**Fig. 1:**
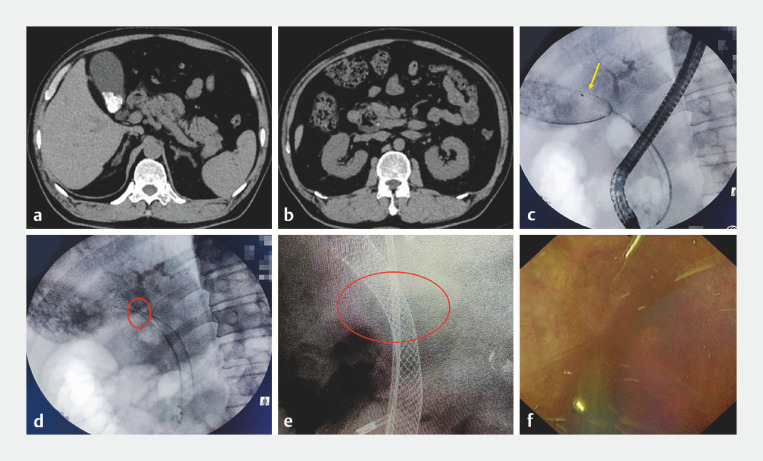
**a, b** Computed tomography images showing multiple gallstones ( **a** ) and choledocholithiasis ( **b** ). **c** A fully covered metal stent was inserted into the neck of the gallbladder (yellow arrow indicates the tip of the stent). **d** A metal stent was used to dilate the narrow cystic duct (red circle). **e** The metal stent is seen in the cystic duct 3 days after dilation. **f** After the gallstones were removed, the gallbladder cavity was irrigated with normal saline under guidance of the eyeMax direct visualization system.

Gallstones were extracted using a basket and a fully covered metal stent under the guidance of the eyeMax direct visualization system.Video 1

The preferred treatment for choledocholithiasis with cholecystolithiasis is ERCP combined with cholecystectomy 1 . However, in certain cases where cholecystectomy cannot be performed, gallbladder-preserving cholecystolithotomy is a safe and effective alternative 2 3 . Extraction of cholecystolithiasis through physiological passages is less invasive. The use of a fully covered metal stent facilitates repeated access to the gallbladder. Additionally, the eyeMax direct visualization system enhances the intuitive nature of the surgery and improves the accuracy of stone collection using baskets. We recommend this procedure for the management of selected patients with gallstones.

Endoscopy_UCTN_Code_TTT_1AR_2AH

## Notes

Endoscopy E-Videos https://eref.thieme.de/e-videos
*E-Videos* is an open access online section of the journal *Endoscopy* , reporting on interesting cases and new techniques in gastroenterological endoscopy. All papers include a high-quality video and are published with a Creative Commons CC-BY license. Endoscopy E-Videos qualify for HINARI discounts and waivers and eligibility is automatically checked during the submission process. We grant 100% waivers to articles whose corresponding authors are based in Group A countries and 50% waivers to those who are based in Group B countries as classified by Research4Life (see: https://www.research4life.org/access/eligibility/ ). This section has its own submission website at https://mc.manuscriptcentral.com/e-videos .
